# Guar Gum Stimulates Biogenic Sulfide Production in Microbial Communities Derived from UK Fractured Shale Production Fluids

**DOI:** 10.1128/spectrum.03640-22

**Published:** 2022-12-01

**Authors:** Lisa Cliffe, Natali Hernandez-Becerra, Christopher Boothman, Bob Eden, Jonathan R. Lloyd, Sophie L. Nixon

**Affiliations:** a Department of Earth and Environmental Sciences, The University of Manchester, Manchester, United Kingdom; b Rawwater Engineering Company Limited, Culcheth, United Kingdom; c Manchester Institute of Biotechnology, The University of Manchester, Manchester, United Kingdom; Swansea University

**Keywords:** bacteria, Bowland, hydraulic fracturing, shale, sulfidogenic, thiosulfate-reducing

## Abstract

Shale gas production fluids offer a window into the engineered deep biosphere. Here, for the first time, we report on the geochemistry and microbiology of production fluids from a UK shale gas well in the Bowland shale formation. The composition of input fluids used to fracture this well were comparatively lean, consisting only of water, sand, and polyacrylamide. This formation therefore represents an interesting comparison to previously explored fractured shales in which more additives were used in the input fluids. Here, we combine cultivation and molecular ecology techniques to explore the microbial community composition of hydraulic fracturing production fluids, with a focus on the potential for common viscosity modifiers to stimulate microbial growth and biogenic sulfide production. Production fluids from a Bowland Shale exploratory well were used as inocula in substrate utilization experiments to test the potential for polyacrylamide and guar gum to stimulate microbial metabolism. We identified a consortium of thiosulfate-reducing bacteria capable of utilizing guar gum (but not polyacrylamide), resulting in the production of corrosive and toxic hydrogen sulfide. Results from this study indicate polyacrylamide is less likely than guar gum to stimulate biogenic sulfide production during shale gas extraction and may guide planning of future hydraulic fracturing operations.

**IMPORTANCE** Shale gas exploitation relies on hydraulic fracturing, which often involves a range of chemical additives in the injection fluid. However, relatively little is known about how these additives influence fractured shale microbial communities. This work offers a first look into the microbial community composition of shale gas production fluids obtained from an exploratory well in the Bowland Shale, United Kingdom. It also seeks to establish the impact of two commonly used viscosity modifiers, polyacrylamide and guar gum, on microbial community dynamics and the potential for microbial sulfide production. Not only does this work offer fascinating insights into the engineered deep biosphere, it could also help guide future hydraulic fracturing operations that seek to minimize the risk of biogenic sulfide production, which could reduce efficiency and increase environmental impacts of shale gas extraction.

## INTRODUCTION

Shale gas is considered a bridge fuel between fossil fuels and renewables, owing to lower CO_2_ emissions upon its combustion compared with coal (1, 2). Shale gas exploitation involves drilling a vertical well down into the shale rock formation, followed by a horizontal well to maximize formation contact. This drilling process is followed by hydraulic fracturing. During hydraulic fracturing, large volumes of water-based fluid are pumped downwell at high pressures to induce fractures in the shale formation and liberate natural gas (3, 4). Following hydraulic fracturing, a proportion of these fluids return to the well pad surface; early fluids are known as “flowback” while later fluids that are thought to be more reflective of the formation itself than the input fluids are known as produced waters (5 to 8). These fluids offer valuable insights into the chemistry and microbiology of fractured shale (9, 10) and will be referred to as “produced fluids” collectively. Prior studies suggest that hydraulic fracturing can create new ecosystems in the deep subsurface (10, 11). Analysis of shale gas production fluids indicates that fractured shales harbor adapted and active microbial communities, despite the common addition of biocides to input fluids (7, 9, 10, 12, 13). The fermentative halophile Halanaerobium frequently dominates shale gas production fluids spanning geographically distinct wells across North America (5, 7, 9 to 19) and are capable of reducing thiosulfate to sulfide (20, 21). Biogenic sulfide production is a major concern to the oil and gas industry as sulfide gas is hazardous to human health and highly corrosive and can “sour” natural gas, making it costly to refine (22–24). Previous studies have postulated that commonly used fracturing fluid additives may stimulate biogenic sulfide production under relevant conditions (25).

Hydraulic fracturing fluids are predominantly composed of water and sand, along with a variety of other additives (26–28). Viscosity modifiers are used in all conventional fracturing operations, with guar gum representing the most widely disclosed viscosity modifier used for gel-based hydraulic fracturing (26). An alternative method, “slickwater fracturing,” uses friction reducers instead, such as polyacrylamide (29, 30). Other additives, including corrosion and scale inhibitors, antiswelling agents, clay stabilizers, and biocides are also typically added to make the process more efficient (26, 28, 29). The use of fracturing additives varies between wells, and their disclosure (via databases such as fracfocus.org) is largely voluntary in most countries. In the United States, dozens of fracturing additives are typically used in input fluids (29, 31, 32). In contrast, a relatively lean input fluid chemistry was approved for exploratory drilling of the Bowland Shale, United Kingdom, which involved using only water (treated with UV sterilization), sand, and up to 0.05% wt/vol polyacrylamide as a friction reducer.

The fate of fracturing chemicals downwell is largely unknown, yet a large number of these chemicals are known to be biodegradable (17, 26, 29, 31). The commonly used gelling agent guar gum is a high molecular weight polysaccharide made up of galactose and mannose sugars, and is presumed to be readily biodegradable (25, 26, 29). This additive has been linked previously to sulfide generation at elevated pressures through the activity of fresh-water-derived sulfate-reducing bacteria (SRB) (25). Further, the microbial degradation of guar gum by *Halanaerobium* strain DL-01 has been linked to the production of acetate and sulfide (18). The microbially enhanced anaerobic degradation of hydrolyzed polyacrylamide, a common friction reducer, has also been demonstrated in enrichments from oil filed produced waters (33), and SRB have been previously shown to utilize hydrolyzed polyacrylamide as the sole carbon source (34). Previous reports also suggest polyacrylamide facilitates the dissolution of minerals such as pyrite (a common component of shale), leading to the release of iron and sulfate (30). The incomplete oxidation of pyrite, such as in the anoxic environment of fractured shale (35), could lead to the generation of intermediate sulfur species, including thiosulfate, which could serve as an electron acceptor during anaerobic respiration (36). Given the corrosive and toxic nature of hydrogen sulfide, it is important to consider the souring potential of fractured shale microorganisms that can couple the degradation of common fracturing fluid additives, including the viscosity modifiers considered here, to the reduction of a range of sulfur species.

Here, microbiological data from production fluids obtained from an exploratory shale gas well in the Bowland Shale (UK) are reported. We utilized culture-based approaches, geochemical analysis, and molecular ecology techniques to give a broad picture of microbial ecology and metabolism in the fractured Bowland shale formation. Hydraulic fracturing production fluids were collected from a well designated “Bowland-1,” and their geochemistry and microbiology characterized prior to their use as inocula in substrate utilization tests. The objectives of this work were to (i) assess production-fluid microbial communities in the Bowland shale and (ii) evaluate the potential for two commonly used viscosity modifiers (guar gum and polyacrylamide) to stimulate sulfide production by sulfate-reducing and thiosulfate-reducing bacteria derived from these production fluids. This work offers a useful comparison of microbial communities in UK shale gas formations, and could guide future fracturing decisions.

## RESULTS

### Production fluid geochemistry.

Overall salinity in Bowland-1 production fluids ranged from 47,500 mg/L to 79,800 mg/L chloride (Table 1). No thiosulfate, nitrate, nitrite, or volatile fatty acids (VFAs) were detected in any production fluid samples. On average, samples contained 113.2 mg/L sulfate and ranged from 111.4 mg/L to 120.1 mg/L (Table 1). Concentrations of bromide, chloride, potassium, magnesium, and strontium generally increased with time since hydraulic fracturing began. We observed a general decrease in total organic carbon and a general increase in salinity, over time (Table 1).

**TABLE 1:** Production fluid geochemistry^*a*^

	Days since HF							
Analyte	60	62	65	67	68	81	82	86
pH	6.2	6.3	6.3	6.2	6.3	60	6.1	6
TOC	10.3	8.8	9.3	8.7	15.1	6.7	7.2	6.8
Chloride	54,883.6	49,025.3	47,466.5	51,831.6	47,622.8	73,932.5	79,823.3	76,776.8
Bromide	407.8	384.9	396	434.3	392.9	636.3	686.9	652.9
Sulphate	111.4	113.9	112.8	119.3	101.8	115	120.1	111.5
Sodium	12,589.3	13,855	12,474.6	9,260.8	14,953	20,339.4	15,834.6	17,575.1
Magnesium	798.2	804.3	783.9	523.4	866.3	1.490.9	1,122.2	1,304.1
Silicon	n.d	8.1	13.8	19.1	29.8	23.9	22.6	24.1
Potassium	34.9	16.6	29.3	30.1	30.7	43.3	37.8	42.5
Calcium	1,662	1,729.3	1,744.9	1,202	1,929.7	3,295.9	2,514.5	2,899.4
Manganese	23.3	1.7	1.2	0.7	1.1	1.5	1.7	1.3
Iron	43.6	18	19.8	14	21.8	30.8	23.2	30.9
Copper	22.8	0.4	n.d	0.1	n.d	0	n.d	n.d
Zinc	34.2	2	47.8	4.6	2.6	1.8	4.9	4.8
Strontium	837.6	871.1	859.6	590.5	953.1	1,632.6	1,233.6	1,432
Barium	28.2	6.4	5.5	3.6	6.4	8.3	6.1	7.6
Lead	22.8	0.8	0.4	0.5	0.3	0.4	0.3	0.4

a HF, hydraulic fracturing; n.d., not detected; TOC, total organic carbon. All values are in mg/L.

### Microbiology of shale gas production fluids.

Production fluid DNA yields were too low for fluorometric quantification (<0.2 ng), so most probable number (MPN) enumerations were used as a means of quantification. Fermentative bacteria were targeted because they are known to dominate fractured shale communities (44). We hypothesized that viscosity modifiers in the enrichments would be degraded via fermentative microorganisms and that the resulting by-products of fermentation would serve as electron donors for sulfate- and thiosulfate-reducing bacteria, resulting in the production of sulfide. MPN data suggested a maximum culturable cell count of 960 fermentative bacterial cells per mL across the latter three time points (Table S1 in the supplemental material).

**FIG 1:**
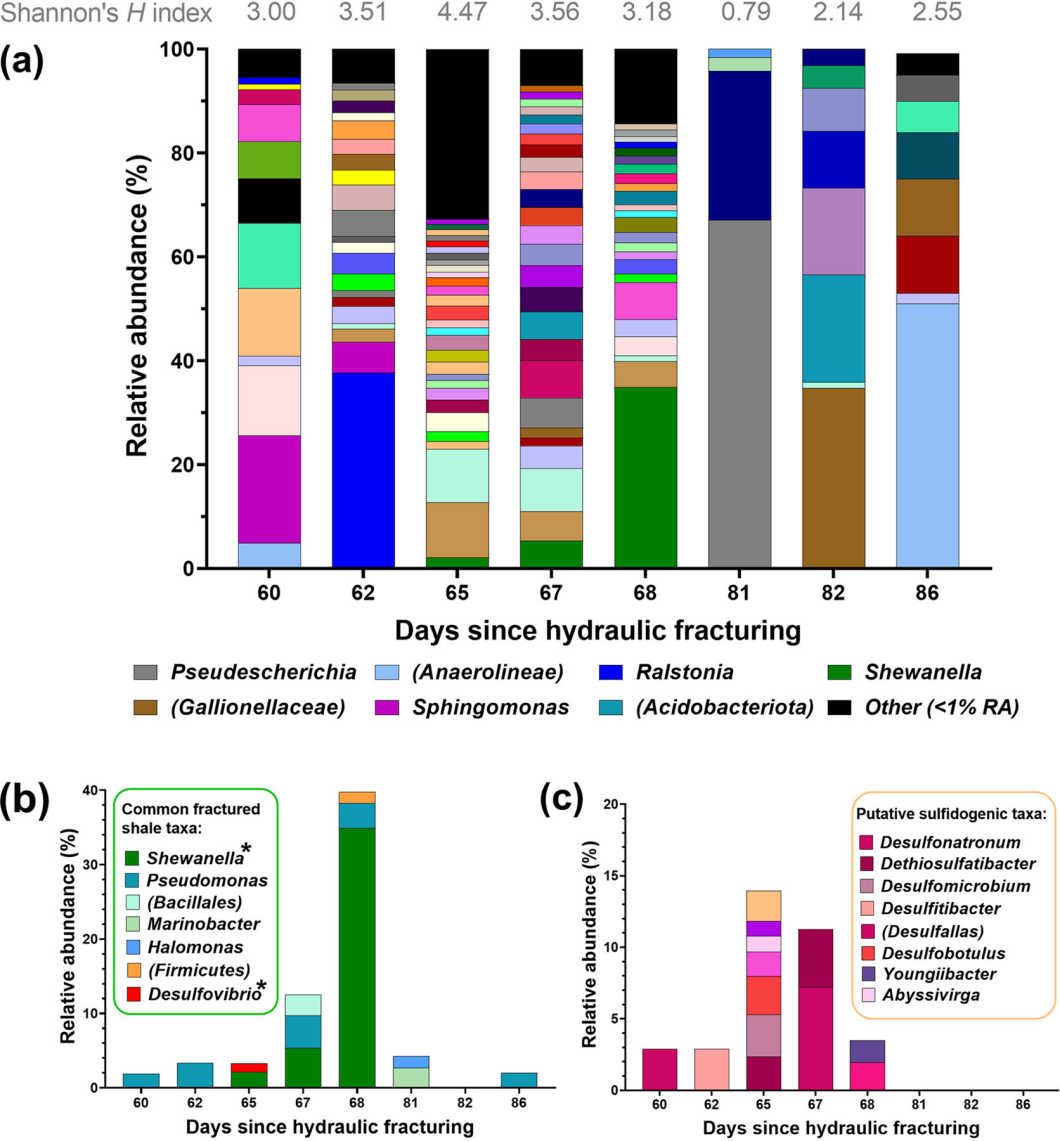
Bacterial community composition of UK-produced waters based on 16S rRNA gene sequencing. Taxa displayed at genus level or the next highest resolved phylogeny in brackets. (a) All taxa, where “other” representing species with a combined relative abundance (RA) below 1%. (b) Commonly detected fractured shale taxa based on previous literature. *, potential sulfidogenic taxa identified here and in previous shale gas-produced water studies. (c) Putative sulfidogenic taxa.

Despite below-detection yields of DNA, we obtained 16S rRNA sequencing data of the microbial communities present in flowback fluids. The microbial community composition of flowback fluids was determined using 16S rRNA gene sequencing. These data indicate a dynamic microbial community that varied from day to day. Samples collected at 62, 65, and 67 days since hydraulic fracturing began were particularly diverse (Fig. 1a). Samples collected 82 and 86 days after hydraulic fracturing showed lower species diversity compared to the earlier time points (Fig. 1a). A number of putative sulfidogenic taxa were detected in the production fluids, including representatives of the genera Desulfovibrio and Shewanella (Fig. 1c). *Desulfovibrio* species are common SRB associated with fractured shale and oil production facilities (9), and were only detected here at 65 days post hydraulic fracturing, at <1.2% relative abundance (RA) (Fig. 1b). Sequences affiliated with the genus *Shewanella* were detected in Bowland shale production fluids at days 65, 67, and 68 at increasingly higher RA (Fig. 1b), and represented the highest overall RA of the day-68 sample (Fig. 1a). Some species of *Shewanella* are capable of thiosulfate reduction (45), and *Shewanella* were reported as the predominant taxa in production fluids from the Sichuan basin, China (46, 47).

### Sulfidogenic enrichments.

**FIG 2:**
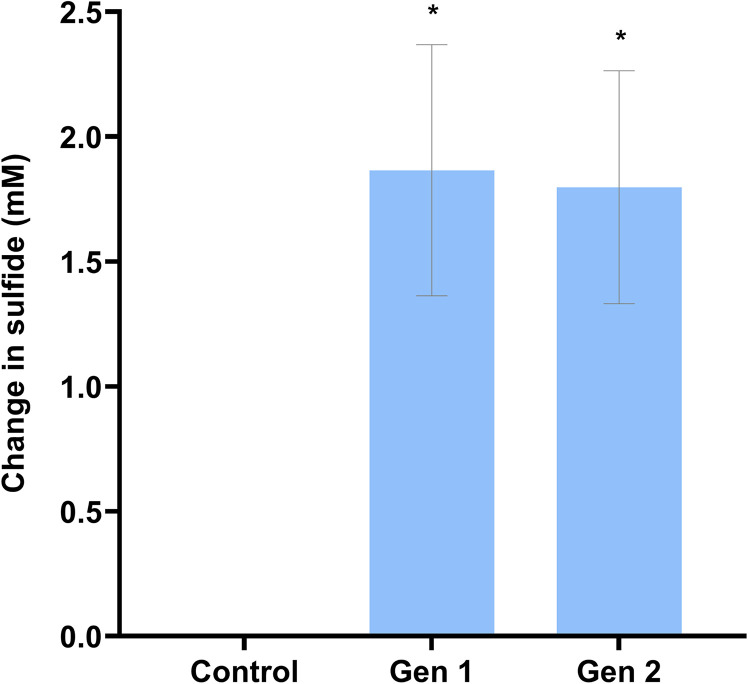
Average change in sulfide (mM) detected in cultures amended with guar gum and thiosulfate. After 150 days (control and gen 1) and 119 days (gen 2). Control shown here is noninoculated minimal medium amended with guar gum with thiosulfate. Error bars show the standard error of the mean. *, statistically significant change (*P* < 0.005).

All enrichments amended with guar gum and thiosulfate underwent a distinct color change from colorless to black, indicative of iron sulfide formation, whereas no other enrichments or controls gave rise to a visible color change (Fig. S1). A measurable concentration of sulfide was detected (after filtration to remove iron sulfide) in the liquid phase of all guar gum-amended thiosulfate enrichments. Further, these enrichments (generations 1 and 2) showed a statistically significant (student’s two-tailed *t* test, *P* < 0.05) increase in sulfide from start to end (*P* = 0.0003 and *P* = 0.0001, respectively). An average of 1.9 mM sulfide was produced from generation 1 (R^2^ > 0.999) after 150 days incubation, and 1.8 mM sulfide (R^2^ > 0.999) from generation 2 after 119 days incubation (Fig. 2). We noted that thiosulfate is a known inhibitor of methylene blue color formation (48), so this could have resulted in lower readings than were truly present. No free soluble sulfide or FeS was detected at the onset of experiments, in any other enrichment tests or in the controls.

### Geochemical analysis of enrichments.

Sulfate and thiosulfate concentrations were monitored using ion chromatography. Sulfate concentration did not decrease over time in any sulfate-reducing enrichments or controls, indicating that no sulfate reduction had occurred. Similarly, there was no significant change in thiosulfate concentration over time in the enrichments amended with polyacrylamide. This indicated that polyacrylamide did not support the reduction of thiosulfate. In contrast, enrichments amended with guar gum and thiosulfate showed a significant decrease in thiosulfate in both generation 1 (*P* = 0.0332) and generation 2 (*P* = 0.0306) enrichments. This observation is concomitant with measurements of sulfide generation observed in the enrichments amended with guar gum and thiosulfate (Fig. 2).

Taken together, these results suggest that biologically mediated reduction of thiosulfate was responsible for sulfide production through the utilization of guar gum (and its degradation products). No significant change in thiosulfate concentrations were seen in the abiotic controls over the course of experiments (*P* = 0.3045), confirming that abiotic thiosulfate reduction did not contribute to sulfide production in these experiments.

**FIG 3:**
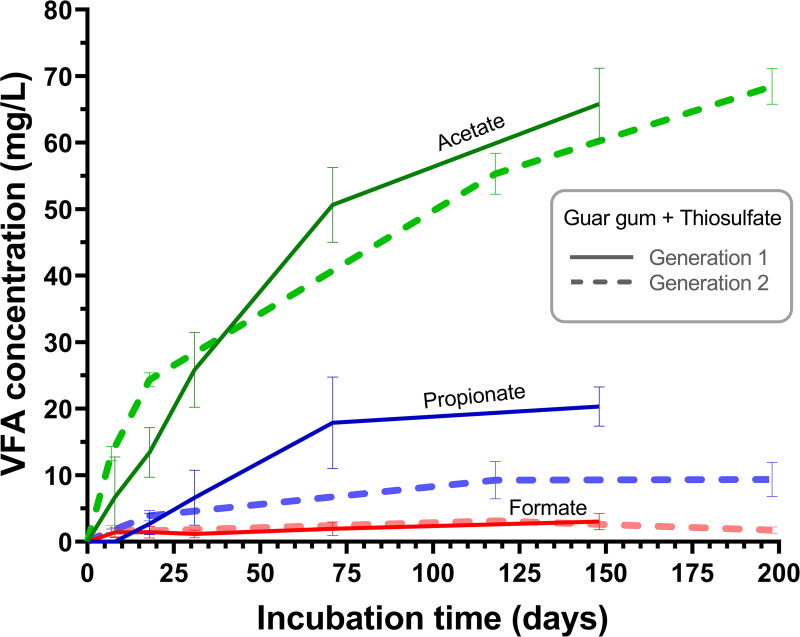
Change in concentration of volatile fatty acids (VFAs) in cultures supplied with guar gum and thiosulfate, based on mean of triplicate data sets. Error bars display standard deviation. Acetate concentrations are displayed in green, propionate in blue, and formate in red. No VFAs were detected in controls. Solid line represents first-generation enrichments, dashed line represents second generation.

Guar gum-amended enrichments gave rise to an increase in VFAs over time in both the thiosulfate- (Fig. 3) and sulfate-amended media (not shown). Guar gum-amended cultures were dominated by acetate, with smaller amounts of propionate and formate (Fig. 3). We suggest that VFAs were intermediates from guar gum biodegradation produced by fermentative microorganisms enriched from the shale production fluids. In contrast, no VFA production was observed in any of the polyacrylamide-amended enrichments or controls.

### Bacterial community composition of viscosity modifier enrichments.

**FIG 4:**
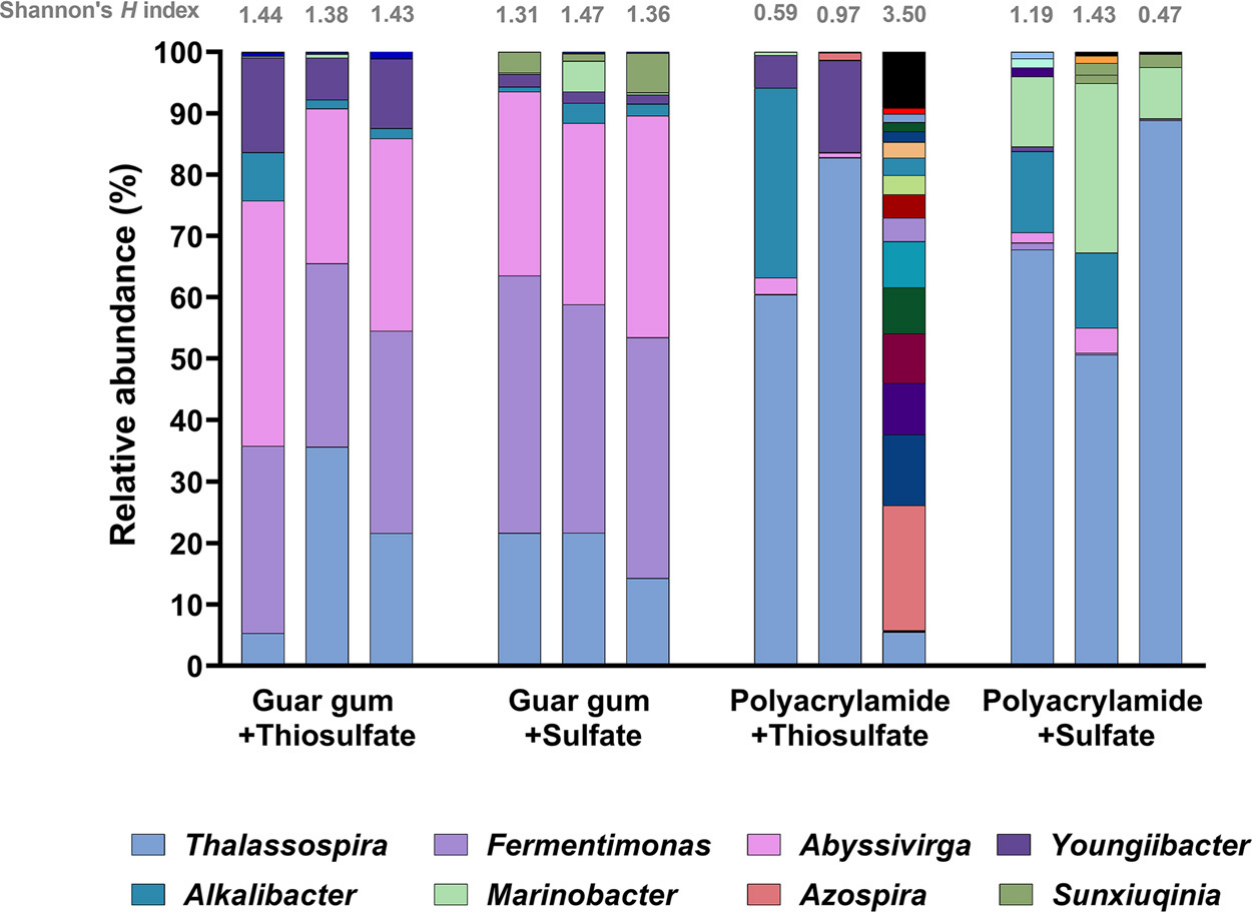
Bacterial community composition of viscosity modifier enrichments based on 16S rRNA gene sequencing of second-generation cultures. Results were taken after 41 days of incubation at 30°C. Shannon’s H index is displayed above each bar. Most dominant genera are indicated in the key.

The microbial community composition of the enrichments was profiled using 16S rRNA gene sequencing (Fig. 4). With the exception of an apparent outlier among one of the polyacrylamide and thiosulfate-amended enrichments, the Shannon diversity indices (Fig. 4) were higher in cultures supplemented with guar gum, suggesting that guar gum stimulated a broader range of microorganisms compared to polyacrylamide.

The guar gum-amended enrichments were dominated by sequences affiliated with Fermentimonas, with relative abundance (RA) ranging from 30% to 42% (Fig. 4). To date, the genus *Fermentimonas* contains only one known species, *F. caenicola*, sharing 97.63% identity over 253 bp with the bacterium identified here (49). Although dominant in the guar gum-amended enrichments, sequences affiliated with *Fermentimonas* were present at <2% RA in the polyacrylamide-amended cultures (Fig. 4) and some initial production fluids (Fig. 1). Another dominant taxon identified in guar gum with thiosulfate enrichments was assigned the genus Abyssivirga. Sequences closely affiliated with *Abyssivirga* were prominent in all guar gum-amended cultures (between 25 and 40% RA) yet were similarly only present at relatively low levels (~5% RA) in the polyacrylamide-amended enrichments (Fig. 4). Representatives of the *Abyssivirga* genus were also detected at 1.1% RA in the produced fluid sample collected 65 days after hydraulic fracturing (Fig. 4). Sequences affiliated with the genus Youngiibacter were detected at higher RA in cultures grown with thiosulfate (up to 15%), compared to sulfate (up to 2%). Conversely, sequences affiliated with the Marinobacter genus were observed at higher RA in sulfate-amended enrichments (up to 28%), compared to thiosulfate (up to 0.6%). In addition, *Alkalibacter* species, a frequently reported taxon recovered from shale gas production fluids (12, 46), were identified in some enrichments (Fig. 4) despite being below the limit of detection in flowback fluids. The microbial profiles of the polyacrylamide-amended enrichments contrasted with the guar gum enrichments, with a lower RA of the Fermentimonas and *Abyssivirga* species, while ASVs closely affiliated with the genus Thalassospira were most heavily represented (Fig. 4). Finally, ASVs affiliated with genera Sphingomonas, Levilinea, and Methanosarcina were detected in the initial production fluids and again in the fracturing chemical enrichments. However, the vast majority of taxa observed in the initial production fluids did not appear to be stimulated by either guar gum or polyacrylamide under the imposed conditions.

## DISCUSSION

### Bowland shale production fluid geochemistry and microbiology.

Overall salinity levels in production fluids recovered from Bowland-1 were lower than those commonly reported in the Marcellus basin, United States (7, 50), but higher than was reported in the Sichuan basin, China (46). Salinity did follow the frequently reported trend of increasing with time since the start of hydraulic fracturing, even across the relatively short time period studied here (4, 7, 9–11, 19). Conversely, sulfate levels were higher than those reported for the Marcellus and Sichuan basins (7, 46, 50). No VFAs were detected in Bowland-1 production fluids, potentially indicating low metabolic activity *in situ*. This assumption was supported by MPN counts that suggested a maximum of 960 cells per mL across the latter three time points. The scarcity of microbial DNA extracted from the production fluids could be a testament to the success of the UV sterilization process that input fluids underwent in place of biocide treatment. The differences in geochemical factors between the production fluids studied here (Table 1) and those previously reported in other formations, could also go some way to explaining the difference in microbiology observed here (Fig. 1).

This study provides the first insights into the microbiology and geochemistry of an exploratory shale gas well drilled and hydraulically fractured in the United Kingdom, and offers the opportunity to compare data with those already studied. Production fluids from the Sichuan basin in China were characterized by lower temperatures, circumneutral pH, and lower salinity compared to its U.S. counterparts (46, 51). Like the Bowland shale, the Sichuan basin was also fractured using slickwater fracturing involving water, sand, and polyacrylamide as a friction reducer (46). However, unlike the Bowland shale, other fracturing chemicals were also used (46). Production fluids from the Sichuan basin were colonized by species of *Shewanella*, *Marinobacter*, Desulfovibrio, and Dethiosulfatibacter, and these genera were also detected in the Bowland-1 production fluids (Fig. 1). However, the microbial communities identified in Bowland-1 production fluids bore little resemblance to those previously reported in the United States and Canada, as shown by the low RA of commonly reported shale taxa (Fig. 1b). The lack of sequences affiliated with the commonly dominant fractured shale taxa, *Halanaerobium*, in the Bowland-1-well indicates that the equivalent niche is dominated by another lineage downwell. We propose that the major changes in microbial community composition observed in fractured Bowland shale production fluids compared to previous studies are driven primarily by the comparatively lean input fluid composition, as well as the lower salinity.

### Bioavailability of viscosity modifiers.

Given that Bowland-1 was fractured via slickwater fracturing, with polyacrylamide as the added viscosity modifier, the microbial communities recovered from Bowland-1 production fluids had likely not encountered guar gum previously. However, our results indicate that guar gum was able to stimulate microbial communities and support the microbial reduction of thiosulfate, leading to sulfide production (Fig. 2). Guar gum consists of galactose and mannose sugars, is known to be readily biodegradable (25, 26, 29) and appears to be bioavailable to the communities recovered in Bowland-1 flowback fluids. Guar gum degradation yielded a number of volatile fatty acids (VFAs) (Fig. 3), which could in turn support the metabolism of a wider consortium of microorganisms. Numerous reports indicate that polyacrylamide can serve as a carbon/nitrogen source in support of microbial growth (33, 34, 52–54). Previously, the degradation of polyacrylamide has been linked to the production of VFAs (33, 55), acrylic acid, methane (55, 56), and the toxic monomer acrylamide (52, 57). However, no VFA production was observed in any of the polyacrylamide-amended enrichments reported here. Without directly measuring the levels of guar gum and polyacrylamide over the course of these experiments, our conclusions are based on the accumulation of by-products indicative of degradation and microbial community changes when guar gum/polyacrylamide were provided as the sole carbon sources for enrichments. Our results support the hypothesis that polyacrylamide is a comparatively less bioavailable polymer (58) than guar gum. It may be difficult for microorganisms to access the carbon backbone of polyacrylamide given the scarcity of appropriate enzymes compared to the breadth of amidase enzymes able to liberate nitrogen from the amide groups of polyacrylamide (56, 59, 60). As such, while polyacrylamide may serve as a source of nutrients, we infer that microbial communities recovered from Bowland-1 production fluids are not able to use it as a substrate for growth. Indeed, our results suggest that polyacrylamide is therefore less likely than guar gum to stimulate biogenic sulfide production, as its apparent degradation did not support the production of sulfide via microbially mediated sulfate or thiosulfate reduction.

### Putative guar gum degraders and sulfide producers from the Bowland-1 shale gas well.

Members of the *Fermentimonas* genus appeared to dominate guar gum-amended cultures (Fig. 4), and the production of VFAs in these enrichments coupled to their low abundance in polyacrylamide-amended cultures suggests they contributed to guar gum degradation. Sequences affiliated with the genus *Fermentimonas* were observed at low relative abundance (less than 2%) in production fluids and polyacrylamide-amended enrichments, indicative of opportunistic growth in the presence of a substrate they could metabolize. Indeed, previous reports indicate that *Fermentimonas* are capable of fermenting d-galactose and d-mannose (61), the constituent sugars of guar gum (62). We have found no prior reports of the presence of *Fermentimonas* in shale gas production fluids, yet given their dominance in guar gum-amended cultures and the lack of *Halanaerobium* spp., we hypothesize that these bacteria occupy the equivalent niche in our systems to more halophilic fermentative lineages in U.S. fractured shale systems.

Although not detected in initial flowback fluids (Fig. 1), members of the genus *Thalassospira* were detected across all fracturing chemical enrichments (Fig. 4). Members of this genus were present at lower relative abundance in guar gum-amended enrichments compared with polyacrylamide-amended enrichments, yet their relative enrichment in guar gum enrichments compared with flowback fluids suggests they play a role in its degradation. Further, this genus dominates polyacrylamide-amended enrichments (Fig. 4), and while we did not observe the production of sulfide or VFAs in these experiments, their dominance may indicate an active role in polyacrylamide degradation. Members of the genus *Thalassospira* have previously been isolated from a variety of marine environments, including iron-treated seawater, marine sediments, and oil spill-contaminated seawater (63, 64). They are typically halophilic facultative anaerobes, with some species believed to play a role in hydrocarbon degradation, either directly or as part of a microbial consortium (64, 65). Further, some *Thalassospira* species are capable of biofilm formation that promotes the corrosion of carbon steel under laboratory conditions (66). This corrosive potential, and their apparent enrichment in the presence of common viscosity modifiers, could be of some concern to shale gas producers and is worthy of further study.

Sequences closely related to the genus *Abyssivirga* were prevalent in the guar gum-amended enrichments. One isolated member of this genus, Abyssivirga alkaniphila, grows anaerobically at 14 to 42°C and has a salinity range of 0.5 to 6% NaCl (67), broadly in line with conditions imposed here. Abyssivirga alkaniphila strain L81^T^ has been shown previously to utilize hydrocarbons, sugars, and amino acids for growth, and thiosulfate has been identified as an electron acceptor during the degradation of hydrocarbons (67). This taxon is the most abundant lineage in our substrate utilization experiments that has been previously implicated with thiosulfate reduction (67). When taken together with our results, we infer that members of this genus contributed to the observed sulfide generation (Fig. 3). Abyssivirga alkaniphila was first isolated from a hydrothermal vent in the arctic midocean ridge (67) and has since been detected in low-temperature oil fields in Russia, along with *Halanaerobium* (68). However, members of *Abyssivirga* have not been previously reported in any shale gas production fluids or enrichments to date. Previously, yeast extract was reported to be a requirement for the growth of *Abyssivirga* (67), but enrichments studied here suggest this not to be the case as no yeast extract was present in the media. Since *Abyssivirga* species have the capacity to reduce thiosulfate and degrade hydrocarbons, their presence in the fractured shale environment could be considered a problematic.

Lineages associated with the genus *Youngiibacter* were detected at low levels in the production fluids (Fig. 1c) and again across the fracturing chemical enrichments (Fig. 4), with relative abundances appearing to increase in the presence of thiosulfate. Previous studies have reported that Youngiibacter fragilis was able to utilize galactose and mannose, the constituent sugars that make up guar gum, and is also capable of thiosulfate reduction (69). This bacterium was originally isolated from natural gas produced waters with an optimum growth temperature of 15 to 36°C and salinity tolerance of up to 5% NaCl (69), again broadly in line with the conditions imposed here. Further work is required to establish the true role of *Youngiibacter* in hydraulically fractured shale gas production facilities.

**Conclusions.**We demonstrated that the addition of guar gum in the presence of thiosulfate led to the measurable production of sulfide by a microbial community recovered from a UK shale gas well. Both viscosity modifiers appeared to enrich for fermentative lineages, but only guar gum-amended enrichments supplied with thiosulfate (not sulfate) gave rise to the production of sulfide. Microbial community composition analysis and within the context of previous literature suggests that organisms such as *Abyssivirga* and *Youngiibacter* may potentially be responsible for the thiosulfate reduction reported in the guar gum-amended enrichments, in contrast to previous studies where *Halanaerobium* was identified as the predominant taxon with sulfidogenic capabilities (6, 9, 18, 70). Indeed, our findings highlight several lineages not previously associated with fractured shale microbial communities, which we infer is attributable to the lower salinity of production fluids coupled to the lean composition of input fluids used to fractured this exploratory well.

Guar gum is one of the most commonly used viscosity modifiers in hydraulic fracturing operations, owing largely to its low cost. However, our findings highlight the potential for this additive to stimulate microbial growth leading to the production of sulfide, which could have significant implications for the recovery of shale gas and the lifetime of the wells. In contrast, our results indicate that polyacrylamide, the most commonly used additive in slickwater fracturing operations, is relatively less bioavailable and may the better choice to avoid sulfidogenesis. Further study is required to assess the potential for these additives to stimulate deleterious microbial growth under downwell conditions. Assessing the potential for common fracturing additives to stimulate microbial growth and metabolism, as we have demonstrated here, may help inform best practices to achieve the most efficient and cost-effective and least environmentally damaging approach to shale gas extraction.

## MATERIALS AND METHODS

### Sample collection.

Production fluids were collected from the gas-water separator of a newly drilled exploratory shale gas well designated “Bowland-1” in the North of England, United Kingdom, using sterile polyethylene bottles filled to capacity to exclude oxygen. Samples were collected 60, 62, 65, 67, 68, 81, 82, and 86 days after hydraulic fracturing began. Samples were transported on ice to the University of Manchester, where aliquots were taken anaerobically for use in culturing. The remaining sample volume was frozen for future geochemical and DNA-based analyses. Anaerobic aliquots were stored at room temperature in the dark.

### Geochemical analysis.

Total organic carbon (TOC) concentrations in the liquid phase of production fluids were measured with the high-temperature catalytic oxidation method (680°C) using a TOC analyzer (Shimadzu TOC-VCPN, Japan). Supernatants (600 μL) from samples that had been centrifuged at room temperature (14,000 *g* for 7 min) were diluted 25-fold and introduced to the TOC analyzer using an autosampler (ASI-V, Shimadzu, Milton Keynes, UK). Concentrations of total carbon and inorganic carbon were obtained from five-point potassium hydrogen phthalate and sodium carbonate standard calibration curves, respectively. Total organic carbon was calculated by subtracting the inorganic carbon from total carbon measurements. Other analytes (Na, Mg, Si, K, Ca, Mn, Fe, Cu, Zn, Sr, Ba, and Pb) were measured by acidifying the liquid phase of production fluids in 2% vol/vol HNO_3_ and analyzed by inductively coupled plasma atomic emission spectroscopy (ICP-AES, Perkin–Elmer Optima 5,300 DV). Production fluids and enrichments were screened for anions using ion chromatography (IC). Concentrations of chloride, nitrite, nitrate, bromide, sulfate, thiosulfate, and selected volatile fatty acids were measured using a Dionex ICS5000 (Thermo Scientific) alongside appropriate standards (Sigma-Aldrich).

### DNA extraction, PCR amplification, and 16S rRNA gene sequencing.

Samples were stored at −80°C prior to analysis. For DNA extractions, biomass was concentrated by centrifugation to maximize DNA yields. Specifically, 400 mL aliquots were centrifuged at 10,000 *g* for 20 min to obtain pellets that were resuspended in 5 mL supernatant. For enrichment cultures, DNA was extracted without concentration. All enrichment-based extractions were conducted using the DNeasy PowerLyzer PowerSoil kit (Qiagen, Manchester, United Kingdom) using 0.2 mL subsamples following the standard protocol supplied by the manufacturer. All extraction runs were performed with two negative extraction controls. Double-stranded (ds) DNA was quantified using the Qubit dsDNA High Sensitivity (HS) assay according to manufacturer’s instructions (Invitrogen), and the concentration of dsDNA was read using a Qubit Fluorometer (Thermo Fisher Scientific) with a detection range of 0.2 to 100 ng. PCR amplification included positive (Escherichia coli) and negative (ultrapure water) controls. Sequencing of PCR amplicons of the 16S rRNA gene was conducted with the Illumina MiSeq platform (Illumina, San Diego, United States) targeting the V4 hyper variable region (forward primer, 515F, 5′-GTGYCAGCMGCCGCGGTAA-3′; reverse primer, 806R, 5′-GGACTACHVGGGTWTCTAAT-3′) for 250-bp paired-end sequencing (37, 38). PCR amplification was performed using Roche FastStart High Fidelity PCR System (Roche Diagnostics Ltd., Burgess Hill, United Kingdom) in 50-μL reactions under the following conditions: initial denaturation at 95°C for 2 min, followed by 36 cycles of 95°C for 30 s, 55°C for 30 s, 72°C for 1 min, and a final extension step of 5 min at 72°C. PCR products were purified and normalized to ~20 ng each using the SequalPrep Normalization kit (Fisher Scientific, Loughborough, United Kingdom). PCR amplicons from all samples were pooled in equimolar ratios. Sequencing runs were performed using a 4-pM sample library spiked with 4 pM PhiX to a final concentration of 10% in accordance with Kozich et al. (39). Illumina MiSeq reads were processed using the Quantitative Insights into Microbial Ecology 2 (QIIME2, version 2020.11) pipeline (40). Demultiplexed paired-end sequences were imported to QIIME2 followed by denoising and chimera removal using the Divisive Amplicon Denoising Algorithm 2 (DADA2) plugin (41). QIIME2 feature-classifier “classify-sklearn” (42) was used for the taxonomic annotation of high-quality amplicon sequence variants (ASVs) against the SILVA 138 database (Silva-138-99-515-806-nb-classifier.qza). Both sequencing reaction mix controls and extraction controls were sequenced alongside field and experimental samples, and any shared ASV’s found to be present in the former were manually deleted from ASV data prior to final analysis.

### Fermentative bacteria (FB) medium.

FB medium contained (in g per L deionized water unless otherwise stated) NaCl (80), MgCl_2_.6H_2_O (6.0), NH_4_Cl (2.0), CaCl_2_.2H_2_O (0.4), K_2_HPO_4_ (0.4), KCl (1.5), yeast extract (0.2), d-glucose (2.0), l-Cysteine-HCl-H_2_O (0.3), Na_2_CO_3_ (0.5), resazurin (0.001), and vitamin solution (5 mL). The vitamin solution contained (in mg per L deionized water) biotin (2.0), folic acid (2.0), pyridoxine-HCl (10.0), riboflavin (5.0), thiamine (5.0), nicotinic acid (5.0), pantothenic acid (5.0), vitamin B-12 (0.1), p-aminobenzoic acid (5.0), and thioctic acid (5.0). The medium was dispensed into glass serum vials, purged with an 80:20 mix of N_2_:CO_2_ for 20 min and autoclaved at 121°C for 20 min.

### Minimal medium.

The medium used in substrate utilization tests contained (in grams per L deionized water) NaCl (35), NaHCO_3_ (2.5), NH_4_Cl (0.25), NaH_2_PO_4_.H_2_O (0.6), KCl (0.1), KH_2_PO_4_ (0.5), MgCl_2_.6H_2_O (3.0) along with 10 mL vitamin solution (described above) and 10 mL mineral solution. The mineral solution contained (in mg per L deionized water) nitrilotriacetic acid (1.5), MgSO_4_ (3.0), MnSO_4_.H_2_O (0.5), NaCl (1.0), FeSO_4_.7H_2_O (0.1), CaCl_2_.2H_2_O (0.1), CoCl_2_.6H_2_O (0.1), ZnCl_2_ (0.13), CuSO4.5H_2_O (0.01), AlK (SO_4_)_2_.12H_2_O (0.01), H_3_BO_3_ (0.01), NaMoO_4_ (0.025), NiCl_2_.6H_2_O (0.024), and NaWO_4_.2H_2_O (0.025). Guar gum (0.5 g/L) or polyacrylamide (0.5 g/L) was added to the minimal medium prior to being dispensed, purged, and autoclaved as described above. After autoclaving, either sodium sulfate or sodium thiosulfate was added as terminal electron acceptor from sterile anoxic stock solutions to a final concentration of 17.2 mM. Sterile anoxic iron tetrachloride stock solution was added to a final concentration of 1.72 mM to serve as a visual diagnostic for the production of sulfide, where sulfide reacts with the Fe(II) to form black iron sulfide (FeS) precipitate (Fig. S1).

### Microbial enumeration studies.

Most probable number (MPN) estimates were conducted in FB medium for the latter three production fluid samples (collected 81, 82, and 86 days after hydraulic fracturing) within a week of sample collection. MPN enumerations were used to estimate the number of viable fermentative bacteria able to grow at 8% salinity (reflective of production fluid salinity for these time points). A 10-fold dilution series ranging from 10^−1^ to 10^−7^ was set up for each sample in triplicate and incubated at 30°C, in line with previously reported temperatures (5), and scored after 30 days of incubation. A visual increase in turbidity compared to the negative controls was attributed to microbial growth, and such cultures were scored positive.

### Substrate utilization experiments.

The minimal medium (described above) was designed to assess whether commonly used viscosity modifiers guar gum and polyacrylamide could stimulate sulfide production in hydraulic fracturing production fluid microbial communities from fluids recovered from Bowland exploratory shale gas wells. Guar gum or polyacrylamide was added to achieve a final concentration of 0.05% wt/vol, in line with concentrations commonly used in practice (26, 29). Enrichments were inoculated with a 5% vol/vol mixture containing equal parts of each production fluid. Experiments were run in triplicate alongside substrate-free, abiotic, and heat-treated controls. All enrichments were subcultured into fresh medium (5% vol/vol) after 31 days incubation at 30°C.

### Methylene blue assay.

Aqueous sulfide was measured using the methylene blue assay, adapted from Fonselius et al. (43). Briefly, 1-mL filtered anaerobic subsamples were fixed with 66.7 μL zinc acetate solution (0.15 g zinc acetate dissolved in 10 mL deionized water). Samples were then diluted to within the detection range, and 20 μL mixed reagent containing equal parts N,N-dimethylphenylenediamine dihydrochloride solution (0.2 g dissolved in 10 mL deionized water) and iron chloride solution (0.3 g FeCl_3_ in 10 mL 6M HCl) was added. Radiello RAD171 standards were diluted according to manufacturer’s instructions (Sigma-Aldrich) and used to obtain a standard curve representative of a range of sulfide concentrations at 665 nm. Samples were incubated in the dark at room temperature for 2 h prior to absorbance readings being taken. The standard curve was then used to calculate the equivalent sulfide concentration present in the samples. Sulfide measurements reported in this study were calculated by combining the maximum sulfide in the iron sulfide precipitate (1.72 mM based on a 1:1 ratio of ferrous iron and sulfide in FeS) and the concentration of dissolved sulfide measured using the methylene blue assay (Table S2).

### Data availability statement.

The data sets generated for this study can be found in NCBI SRA under the project accession number PRJNA803344.
