# Supplementary material for: Guar Gum Stimulates Biogenic Sulfide Production in Microbial Communities Derived from UK Fractured Shale Production Fluids
Source: Microbiol Spectr. 2022 Dec 1;10(6):e03640-22. doi: 10.1128/spectrum.03640-22 (PMC9769687; doi:10.1128/spectrum.03640-22)
Supplement: Supplemental file 1 — Supplemental material. Download spectrum.03640-22-s0001.pdf, PDF file, 0.2 MB [file spectrum.03640-22-s0001.pdf]

Supplementary material: Guar gum stimulates biogenic sulfide production in microbial communities derived from UK fractured shale production fluids

Table S1: Results of Most probable number (MPN) estimates performed on fresh production fluid samples. Scored after 30 days incubation at 40°C. “CI” denotes Confidence limits. Column 1 denotes number of days after hydraulic fracturing took place.

Sample	MPN (cells/ml)	Log_{10} MPN	SD log_{10} MPN	95% CI Lower	95% CI Upper	Rarity Index
Day 81	$2.3x10^2$	2.4	0.31	$5.5 x10^2$	$9.6 x10^3$	1.000
Day 82	$2.3x10^1$	1.4	0.31	$5.5 x10^1$	$9.6 x10^2$	1.000
Day 86	$9.2x10^2$	3.0	0.32	$2.1x10^2$	$3.9 x10^3$	1.000

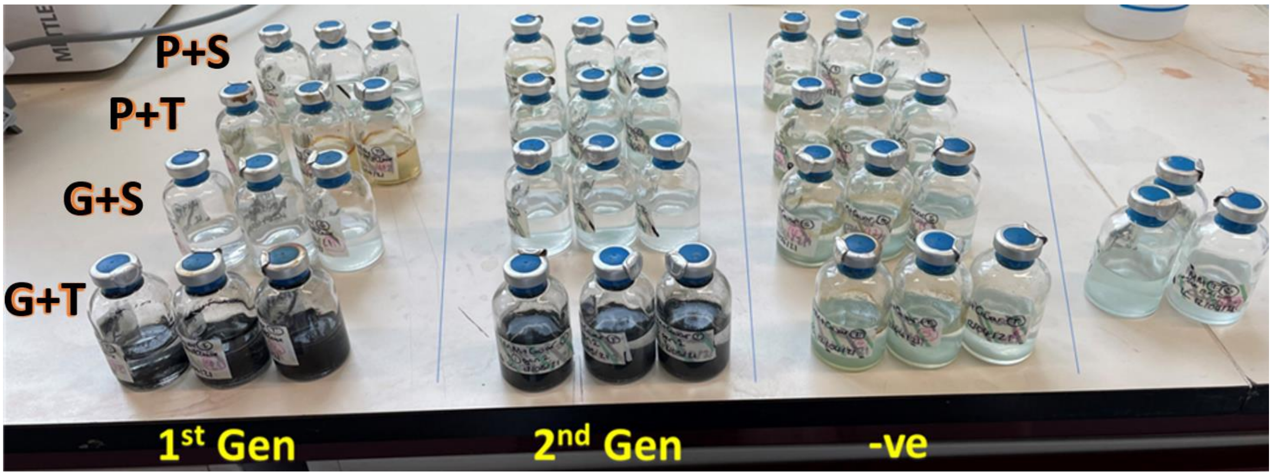

Figure S1: Representative image of fracturing chemical enrichments. Working from bottom row upwards labels correspond with (G+T): Guar gum+ Thiosulfate, (G+S) Guar gum + Sulfate, (P+T) Polyacrylamide + Thiosulfate, (P+S) Polyacrylamide + sulfate. From left to right first 3 rows are 1st generation cultures inoculated with a 5% v/v mix of produced waters, second 3 rows are 2nd generation cultures inoculated from 5%v/v 1st generation culture, third row of 3 are abiotic controls of the same media, final 3 bottles to left are heat-killed controls. The black precipitate seen in G+T cultures is indicative of sulfide generation.

Table S2: Sulfide production in cultures supplied with Guar gum and thiosulfate. "gen 1" denotes the first generation inoculated with 5% v/v produced waters, with sulfide measurements taken after 150 days incubation. "gen 2" denotes the second generation, inoculated with 5% v/v "gen 1, with sulfide measurements taken after 119 days incubation". All concentrations reported are in mM.

**Sample identity**	**Sulfide in solution (mM)**	**Sulfide trapped as FeS (mM)**	**Total sulfide (mM)**
Guar + T (gen1)	0.081	1.720	1.801
Guar + T (gen1)	0.195	1.720	1.915
Guar + T (gen1)	0.160	1.720	1.880
Guar + T (gen2)	0.115	1.720	1.835
Guar + T (gen2)	0.056	1.720	1.776
Guar + T (gen2)	0.061	1.720	1.781
Guar + T (abiotic)	0.000	0	0
Guar + T (abiotic)	0.000	0	0
Guar + T (abiotic)	0.000	0	0

Table S3: accession numbers paired with sample descriptions. All sequencing data related to this publication has been deposited in NCBI SRA under bioproject code PRJNA803344

Accession number	Description
SRS11883330	Guar gum and sulfate amended enrichment
SRS11883329	Guar gum and sulfate amended enrichment
SRS11883328	Guar gum and sulfate amended enrichment
SRS11883322	Guar gum and thiosulfate amended enrichment
SRS11883311	Guar gum and thiosulfate amended enrichment
SRS11883310	Guar gum and thiosulfate amended enrichment
SRS11883313	Polyacrylamide and sulfate amended enrichment
SRS11883312	Polyacrylamide and sulfate amended enrichment
SRS11883334	Polyacrylamide and sulfate amended enrichment
SRS11883333	Polyacrylamide and thiosulfate amended enrichment
SRS11883332	Polyacrylamide and thiosulfate amended enrichment
SRS11883331	Polyacrylamide and thiosulfate amended enrichment
SRS11883316	Produced fluid sample collected 60 days after hydraulic fracturing
SRS11883317	Produced fluid sample collected 62 days after hydraulic fracturing
SRS11883318	Produced fluid sample collected 65 days after hydraulic fracturing
SRS11883319	Produced fluid sample collected 67 days after hydraulic fracturing
SRS11883320	Produced fluid sample collected 68 days after hydraulic fracturing
SRS11883321	Produced fluid sample collected 82 days after hydraulic fracturing
SRS11883323	Produced fluid sample collected 86 days after hydraulic fracturing
SRS11883327	Sequencing control
SRS11883326	Sequencing control
SRS11883325	Sequencing control
SRS11883324	Sequencing control
SRS11883315	Sequencing control
SRS11883314	Sequencing control
